# COVID-19 Pandemic, Physical Distancing Policies, and the Non-Profit Sector Volunteer Force

**DOI:** 10.1177/08997640231163782

**Published:** 2023-04-19

**Authors:** Michael Lebenbaum, Claire de Oliveira, Joanne McKiernan, France Gagnon, Audrey Laporte

**Affiliations:** 1 University of Toronto, Ontario, Canada; 2 Canadian Centre for Health Economics, Toronto, Ontario, Canada; 3 University of York, UK; 4 Volunteer Toronto, Scarborough, Ontario, Canada

**Keywords:** COVID-19, physical distancing, volunteerism, demand for volunteer labor, volunteer interest

## Abstract

Although COVID-19-related physical distancing has had large economic consequences, the impact on volunteerism is unclear. Using volunteer position postings data from Canada’s largest volunteer center (Volunteer Toronto) from February 3, 2020, to January 4, 2021, we evaluated the impact of different levels of physical distancing on average views, total views, and total number of posts. There was about a 50% decrease in the total number of posts that was sustained throughout the pandemic. Although a more restrictive physical distancing policy was generally associated with fewer views, there was an initial increase in views during the first lockdown where total views were elevated for the first 4 months of the pandemic. This was driven by interest in COVID-19-related and remote work postings. This highlights the community of volunteers may be quite flexible in terms of adapting to new ways of volunteering, but substantial challenges remain for the continued operations of many non-profit organizations.

## Introduction

Despite the impacts of COVID-19 on paid labor being widely reported (Bell & Blanchflower, 2020), the effects of physical distancing policies on the provision of volunteer labor have received relatively little attention. Research has discussed the number of individuals recruited for COVID-19-related volunteer drives, COVID-19-related volunteering activities, and the characteristics of volunteers (Mak & Fancourt, 2022; Mao et al., 2021; Mo et al., 2022; Sin et al., 2021), with most studies being qualitative analyses of the experiences of volunteers during the COVID-19 pandemic (Cooney & McCashin, 2022; Seddighi et al., 2020; Sengupta & Al-Khalifa, 2022). The impacts of lockdowns on COVID-19 caseloads, employment, and health-related outcomes have been studied extensively (Brodeur et al., 2021), yet few studies have examined the effect of initiating and loosening of COVID-19 restrictions on demand for or interest to volunteer. One study highlighted increased contributions to Wikipedia during lockdowns (Ruprechter et al., 2021), and another reported on the proportion of volunteers increasing, decreasing, or maintaining the same level of volunteering during a lockdown (Mak & Fancourt, 2022). However, the overall advertised demand for volunteers and interest to volunteer over time during the COVID-19 pandemic and impacts of lockdowns have not been studied. We address this by examining the evolution in the interest in and amount and nature of formal job postings by non-profit organizations (NPOs) through the most popular coordination platform in Canada’s largest city. Unlike most prior crises, which tend to be localized and to last for a short term (Rotolo et al., 2015), COVID-19 is a more drawn-out and widespread affair. Therefore, we also contribute to the crisis volunteering literature where there has been relatively limited quantification of changes in overall levels of volunteering (Lim & Laurence, 2015; Penner et al., 2005; Rotolo et al., 2015), and little is known about the demand for or interest by volunteers in long-running crises (Twigg & Mosel, 2017). This article is structured as follows: Section 2 presents a review of the literature on volunteering during times of crisis and how the COVID-19 pandemic may have influenced volunteering. Section 3 discusses the study setting and data, study variables, and statistical analyses. Section 4 presents the primary and secondary analyses. Finally, Section 5 provides a summary and discussion of the study implications.

## Literature Review

Individuals typically respond in crisis times by helping those affected through greater levels of informal and formal volunteering. Penner et al. (2005) found a very large increase in individuals contacting organizations to volunteer after the 9/11 terrorist attacks in the United States, which was primarily driven by crisis-related activities. This response lasted 3 weeks, after which volunteering returned to pre-9/11 levels for the next 3 months (Penner et al., 2005). Rotolo et al. (2015) examined the American home foreclosure crisis and found that greater metropolitan area–level foreclosures had a positive effect on the overall rates of self-reported volunteering (Rotolo et al., 2015). Volunteer responses to natural disasters such as earthquakes and hurricanes are common and frequently consist of both formal organizations such as the Red Cross and informal volunteering such as spontaneous volunteers (Hustinx & Lammertyn, 2004; Strandh & Eklund, 2018; Twigg & Mosel, 2017). These spontaneous volunteers often include family, friends, or neighbors who typically assist at the neighborhood level through a variety of activities (Strandh & Eklund, 2018; Twigg & Mosel, 2017). These responses can be large with upward of 60% to 70% of the population responding and volunteers numbering in the millions (Twigg & Mosel, 2017). Prior studies on crisis volunteerism have tended to examine the nature of the response (Simsa et al., 2019), motivations (Hustinx & Lammertyn, 2004; Marjanovic et al., 2009), or characteristics of participants (Twigg & Mosel, 2017). However, there has been relatively limited quantification of changes in overall levels of volunteering (Lim & Laurence, 2015; Penner et al., 2005; Rotolo et al., 2015), and little is known about responses to long-running crises (Twigg & Mosel, 2017).

The rich literature on crisis volunteerism suggests that there may be an increase in volunteering in response to the COVID-19 pandemic driven by positions that respond directly to the pandemic’s effects. However, the COVID-19 crisis is more widespread and long-lasting than many prior-studied crises. Due to the high risk of transmission, morbidity, and death, COVID-19 likely places greater risks on volunteers than past social-economic crises such as recessions, foreclosure, and refugee crises (Lim & Laurence, 2015; Rotolo et al., 2015; Simsa et al., 2019). However, COVID-19 risks may be comparable to risks in spontaneous volunteering during natural disasters, which are associated with physical injuries and poor mental health (Sauer et al., 2014). These health risks are particularly important for older individuals who volunteer more hours on average (Hahmann et al., 2020). Meanwhile, in contrast to prior crises, physical distancing policies and health risks restrict many of the most common volunteer activities and settings such as hospitals (Pickell et al., 2020), in-person services (e.g., friendly visiting), and events and fundraisers (Hahmann et al., 2020). Economic models of volunteering examine both investment and consumption motives and would predict reduced interest in volunteering due to greater costs caused by health risks and decreased benefits given there may be less enjoyment and gains in human and social capital among a much more restricted selection of volunteer activities and settings (Govekar P. L. & Govekar M. A., 2002). In contrast to other crises, Lim and Laurence (2015) demonstrated that both formal and informal volunteerism decreased during the 2008 to 2009 recession in the United Kingdom, suggesting the recession caused by COVID-19 may have a similar negative influence. COVID-19 and the subsequent recession have been particularly challenging for NPOs who have seen revenues decrease (Lasby, 2021), program cuts (Kim & Mason, 2020), and layoffs of half the number of volunteer managers (Kurek, 2020). There have also been greater costs with the transition to virtual programming and the need to create new programs (Lasby, 2020). These changes in combination with restrictions in the types of feasible volunteer activities and settings would suggest a decrease in demand for volunteers by non-profits (Govekar P. L. & Govekar M. A., 2002).

## Methods

### Setting and Data

Although Canada at the federal level had modest success in enlisting volunteers (i.e., ~35,000–54,000 volunteers), the volunteers from one program (~54,000) were never used (Miller, 2020), and another program was canceled (Press, 2020). There have also been no successful large-scale recruitment initiatives by the provincial government (Ontario) or city (Toronto). Eight months into the pandemic, roughly 60% of Canadian non-profit leaders reported reduced volunteer numbers and hours (Lasby, 2021).

Toronto is Canada’s largest city with over 2.9 million residents (2018), and Volunteer Toronto is Canada’s largest volunteer center. We used Volunteer Toronto’s repository of volunteer position postings advertised online using the YourMembership volunteer system. Non-profit organizations who post positions include registered charities, volunteer-run organizations, public agencies/organizations, and associations/groups (e.g., religious, community, sport clubs, etc.).

Volunteer postings are active for 90 days, after which they can be renewed. Given that the time over which postings accumulated views was not recorded, starting on February 3, 2020, the data on postings were collected (i.e., downloaded) from the online system every 1 to 4 days (mean 1.3 days) to enable calculation of views and the time between subsequent days when the data were collected. The sample included postings that were active (i.e., searchable on the website) for at least 2 days between February 3, 2020, and January 4, 2021. Postings were only included in the sample on active days as inactive posts do not accumulate views.

### Study Variables

#### Dependent Variables

We defined the demand for volunteers as the total number of active postings each day. The number of views between subsequent collections reflects the interest of people in volunteering. The daily number of views was converted into a daily rate by adjusting the number of views for the variable number of hours between data collections (i.e., daily rate = number of views/hours*24). The data are limited as they only measure interest in formal volunteer positions rather than actual volunteer behavior such as the number of recruited volunteers or volunteer hours worked.

#### Primary Independent Variables

The primary independent variables were the initiation and relaxing of physical distancing measures by the provincial government. Following the World Health Organization declaration of COVID-19 as a pandemic on March 11, 2020, a widely publicized lockdown was initiated across Ontario and included the cancelation of in-person classes, closure of city services and non-essential businesses, and restrictions on gatherings to five people (all instituted during March 11–28, 2020) (Patton, 2020; Rocca & Shah, 2020). Subsequently, Toronto went through multiple other stages with different levels of restrictions including Stage 2 (June 24), (least restrictive) Stage 3 (July 31), Modified Stage 2 (October 10), and a second lockdown (November 23). Description of these policies is included in the Supplemental Appendix Table A1.

#### Covariates/Characteristics

On March 19, 2020, Volunteer Toronto implemented a COVID-19-related response to promote remote and COVID-19-related volunteer positions. A category for COVID-19 positions was added in the search function to make COVID-19 positions highly visible. A “COVID-19 Volunteer Response Team” was formed, and members were sent emails every 3 to 7 days with COVID-19 positions. Volunteer Toronto staff also promoted remote volunteer positions in one-on-one phone calls with prospective volunteers. To explore the potential influence of this programmatic response to the pandemic and the response by citizens to engage in COVID-19 volunteerism, we classified positions as COVID-19 or remote. We identified a position as COVID-19 where the primary category was “COVID-19 response” or if the position included “COVID” or “Corona” in the job title. We identified remote posts as positions classified as “Volunteer from home” or if the position included the words “remote” or “from home” in the job title. Weekly COVID-19 hospitalization numbers (City of Toronto, 2021) and 3-month average of unemployment rates (Statistics Canada, 2021) were also included given these factors represent community needs (i.e., perceived problems) (Ziemek, 2006) that can motivate individuals to volunteer for altruistic purposes and to isolate the influence of physical distancing policies from COVID-19 case loads and economic impacts.

We also examined position type categories, which are the category for the position chosen by the individual who created the volunteer posting. Given there were categories with few (i.e., 3% or less) observations, we combined similar categories together as follows (original and revised categories shown in Supplemental Appendix Table A2). Given that COVID-19 positions did not have a separate category listed to further distinguish the type of position, this may have resulted in artificial decreases in views and post numbers across the original (i.e., pre-COVID-19) set of position types. Therefore, we also characterized positions as event assistance, food related, support with errands, non-food-related drivers or movers, social/mental health, and technology positions by using a combination of position type categories and position title keyword searches (Supplemental Appendix Table A3). We also used organization lists to categorize positions as hospital, rehabilitation hospital, or long-term care (LTC) positions (Toronto Central Healthline, 2021; City of Toronto, 2020).

#### Analysis

The number of active postings, average rate of views, and total of the daily rate of views were graphed. The number of active posts and total of the daily rate of views were also graphed and by type of position. Given there was a large day-to-day variability in views and in active posts by type of position, weekly averages were graphed.

Segmented regressions were used (Wagner et al., 2002), which model the number of views a volunteer posting receives as function of changes in level (i.e., assessing immediate changes) and changes in the time trend/slope (i.e., assessing gradual changes) following each of the five physical distancing periods, while controlling for the pre-existing time trend prior to physical distancing being introduced. Models also controlled for the weekly number of COVID-19 cases, monthly unemployment, and day of the week.

Given views are a count, we used Poisson regressions with robust variance estimation clustering on the posting to estimate the segmented regression models. The offset was set to the natural log of the hours between data collections. To model the data longitudinally, we used random-effects Poisson regression and generalized estimating equations (GEEs) with a Poisson specification and autoregressive of Order 2 correlation matrix, with both types of models estimated with robust standard errors. To account for repeated observations on each posting, GEE models adjust the variance with a covariance structure while random-effects models use posting-specific intercepts (Gardiner et al., 2009) providing complementary approaches given their alternative assumptions and interpretations (GEE: effect on average views across all postings; random effects: effect on average views holding the posting random effect fixed) (Gardiner et al., 2009). In a secondary analysis, a separate Poisson GEE model examined the effect of a volunteer posting being a COVID-19 or remote volunteer position controlling for days of the week, unemployment rate, and weekly COVID-19 cases. All analyses were conducted using Stata version 16.1.

## Results

There were a total of 1,709 volunteer position postings active at any point during the study period. Across the 336 days, data were collected (i.e., downloaded) 249 times, with posts having two to 248 active observations (*M*: 51.4) for a total of 87,670 observations.

**Figure 1.**
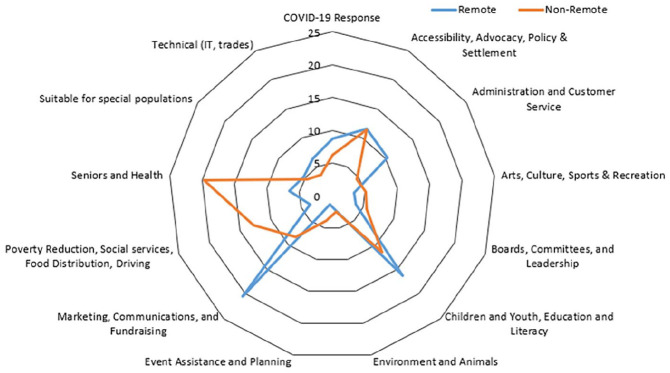
Breakdown of Categories for Remote and Non-Remote Positions.

The most common category for remote positions was the combined category “Marketing, Communications, and Fundraising” (20.5%), while the most common category for non-remote positions was “Poverty Reduction,” “Social Services,” “Food Distribution,” or “Driving” (12.8%) (Figure 1). Of all COVID-19 positions, 73.7% were positions related to “Food” (22.3%), non-food “Driver-Mover” (30.4%), “Social or Mental Health” (11.6%), “Support with Errands” (6.1%), or “Technology” (4.2%) with some overlap across positions.

**Figure 2.**
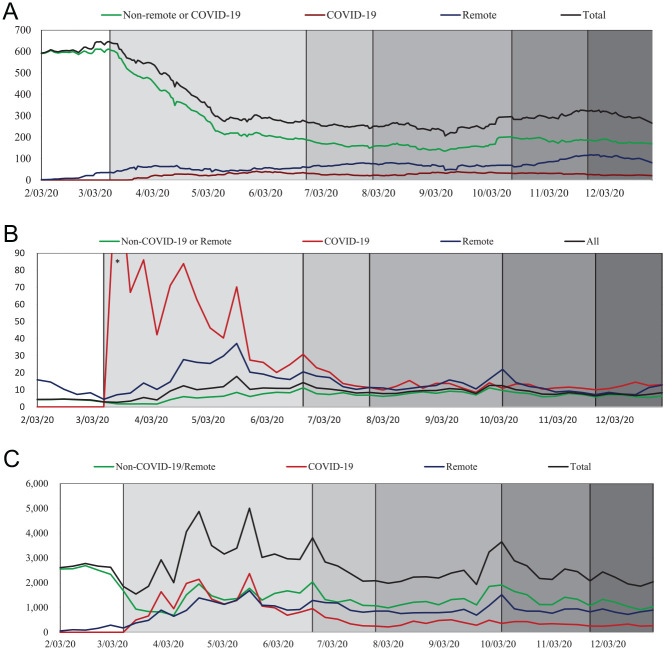
(A) Daily Number of Active Postings. (B) Weekly Average of the Rate Of Posting Views. (C) Weekly Average of Daily Total Rate of Posting Views. *One week had a rate of 149.2.

There was a large decrease in active postings with the onset of physical distancing policies, which was consistently ~50% lower throughout the observation period (Figure 2A). After an initial dip lasting most of March, the daily rates of views and total views increased during the pandemic, and by the end of June, total views returned to and were maintained at pre-physical-distancing levels throughout the rest of the year (Figure 2B/C). The daily rate of views remained elevated throughout the year. The initial increase was largely driven by views of COVID-19 and remote positions, which were highly elevated until late July. There was a large initial decrease in the number of postings (Supplemental Appendix Figure 1A) and total views (Supplemental Appendix Figure 1B) for hospital/LTC and event assistance positions, which continued throughout the rest of the year (Supplemental Appendix Figure 1A). There were large increases in total views for food, driver-movers, social services, and support positions, especially during the first 4 months of the pandemic.

Only the first lockdown period had a change in level that was consistently significant and negative across models, suggesting a large immediate decrease during the first lockdown (Incident Rate Ratio [IRR] range = 0.51–0.73; *p* < .001) (Table 1, Supplemental Appendix Table 4). However, the first lockdown had a significantly positive change in slope, suggesting a gradual increase in views during this period (IRR range = 1.06–1.14; *p* < .001). Overall, periods with more restrictive physical distancing including Stage 2 and Modified Stage 2 were associated with negative changes in slope (IRR range = 0.82–0.93; *p* ≤ .01). The least restrictive physical distancing period, Stage 3, was associated with a positive change in slope in random-effects models only (IRR = 1.07; *p* = .045). There was an approximately 2.66-fold (*p* < .001) increase in views among COVID-19-related positions and 1.8-fold (*p* < .001) increase in remote positions.

**Table 1.** Segmented Regression Analysis of the Effect of Physical Distancing Policies on the Mean Rate of Daily Views.^a^

	GEE Poisson regression w/ robust *SE*	
	Incidence rate ratio	*p* Value
Level changes (i.e., immediate change)		
First lockdown (March 11–June 23)	0.73	<.001
Stage 2 (June 24–July 30)	1.02	.759
Stage 3 (July 31–October 9)	0.61	<.001
Modified Stage 2 (October 10–November 22)	0.86	.020
Second lockdown (November 23–January 4)	0.97	.481
Slope changes (i.e., gradual change)		
Weeks since February 3^b^	1.00	.038
Weeks since start of first lockdown (March 11)^b^	1.06	<.001
Weeks since start of Stage 2 (June 24)^b^	0.93	.003
Weeks since start of Stage 3 (July 31)^b^	1.05	.132
Weeks since start of Modified Stage 2 (October 10)^b^	0.91	<.001
Weeks since start of second lockdown (November 23)^b^	1.01	.644

*Note.* GEE = generalized estimating equations.

a Also controlled for days of the week, unemployment rate, and weekly COVID-19 cases. ^b^Modeled continuously as weeks since the start of the period (and coded 0 prior to that day).

## Discussion

Our findings demonstrate that the COVID-19 pandemic and associated physical distancing has had large impacts on volunteerism in Toronto, Canada. This study found a large (~50%) initial decrease in the number of postings starting with the first lockdown, which remained low throughout the duration of this phase of the pandemic. Although more restrictive physical distancing was generally associated with declining interest in volunteering, during the first lockdown, there were greater total views and a gradual increase in views. Total views remained elevated during the first 4 months of physical distancing restrictions and were driven primarily by large interest in COVID-19-related and remote volunteering positions. Our results highlight the short- and longer-term impacts of COVID-19 and COVID-19-related restrictions, such as lockdowns, on aggregate measures of volunteerism. Furthermore, our results demonstrate that crises can fundamentally change the nature of volunteerism, from in-person to remote, as well as the mix of volunteer organizations and positions available. Finally, they demonstrate, that although long-term demand for volunteers remained similar throughout the year, similar to other crises such as 9/11 (Penner et al., 2005), the volunteer response was relatively short-lived in comparison.

The large shift to COVID-19-related and remote volunteering provides evidence that volunteers are flexible in terms of adapting to new ways of volunteering. The increase in views for COVID-19 positions and COVID-19-related positions such as food distribution, social and mental health roles, and support for errands is similar to that during past natural disasters (Twigg & Mosel, 2017) and past pandemics such as the AIDS pandemic (Omoto & Snyder, 1993). However, this response only lasted approximately 4 months after which total views returned to baseline levels and COVID-19 and remote position views were only slightly elevated relative to other posts. Average views remained elevated throughout the pandemic, but this may be in part because there were approximately half as many posts. Long-term analysis of crises is understudied (Twigg & Mosel, 2017), and our results and a prior study on the 9/11 terrorist attack (Penner et al., 2005) suggest that the volunteer response to some crises might be relatively short-lived. It is not possible to identify the reasons for this short-lived response, but similar experiences with COVID-19-related volunteerism have been described elsewhere (Mao et al., 2021). It is possible that community needs became less apparent when lockdowns were loosened or that these needs may have been addressed to a greater extent by governments (Ziemek, 2006), especially given the large benefits administered by the Canadian government during the pandemic. Alternatively, over time, individuals may have become tired of this emergency, and volunteer efforts may be hard to sustain (Mao et al., 2021). Future, qualitative, and survey-based research should investigate the reasons for these findings.

The introduction of physical distancing measures was accompanied by a large decrease in active positions which points to a significant decrease (~50%) in demand for volunteers by the non-profit sector. This was sustained even with efforts to promote COVID-19/remote positions, additional assistance provided by Volunteer Toronto to NPOs, and with lessening of COVID-19 physical distancing restrictions (i.e., Stage 3), suggesting that the demand may not return to pre-COVID-19 levels until the end of the pandemic. Large decreases in total views and posts for hospitals/LTC institutions suggest that COVID-19 health risks to volunteers and clients likely impeded the ability of non-profits to continue volunteer recruitment. The strict limits on the size of indoor gatherings (5–10 people) throughout much of the pandemic are also likely responsible for decreases in volunteer recruitment given the large and sustained decrease in event assistance volunteering views and posts. These results may also highlight operational challenges that NPOs face, which restrict recruitment of volunteers, such as reduced donations, program cuts, and layoffs of approximately half the number of volunteer managers (Kurek, 2020). Operational challenges have been reported by non-profits in Canada (Lasby, 2020), the United States (Kim & Mason, 2020), and globally (CAF America, 2020), suggesting our results are generalizable beyond Toronto or Canada.

The decreases in demand for volunteers likely resulted in further negative effects of physical distancing for the most vulnerable members of society such as hospital/LTC patients who disproportionately receive services from NPOs. The decrease in opportunities to volunteer may have contributed to unintended negative consequences for individuals who want to volunteer such as lower well-being (Jenkinson et al., 2013). Further policies may be needed to stimulate the demand for volunteers to continue engaging the labor expertise of volunteers, similar to what occurred in the paid employment sector where payments to small businesses by the government were implemented (The Canadian Press, 2020).

Nonetheless, this study is not without limitations. First, the study relied on administrative databases that measure metrics for a large number of units (i.e., thousands of posts among hundreds of organizations) repetitively over time, which is rare among the volunteerism literature and enabled a detailed examination of the influence of different lockdowns, including immediate (i.e., level changes) and delayed (i.e., slope changes). However, the data lacked many variables included in theoretical models of organizational demand for volunteer labor (e.g., volunteer management practices and instruments, organizational attitudes and values, etc.; Studer & von Schnurbein, 2013), which future research should investigate. There was also a lack of qualitative data, which could further explain the findings or provide a deeper understanding of the actions of different NPOs, volunteers, governments, and Volunteer Toronto during different phases of COVID-19 and physical distancing. Collection of qualitative data by future studies such as interviews, analysis of field notes, and document analysis is recommended and will likely help clarify which specific elements of the pandemic, government policies, and programmatic response by the non-profit sector were most impactful and why. Second, volunteer postings were only for formal volunteer positions recruited by non-profits and do not reflect spontaneous volunteering, which has been a common response to past crises such as natural disasters (Twigg & Mosel, 2017) and COVID-19 in some other countries (Monbiot, 2020). Third, the database did not measure the number of people who applied for volunteer positions or completed volunteer hours. However, there is likely a significant relationship between viewing volunteer postings and applying to volunteer positions given 8,000 individuals signed up for the COVID-19 Response team email list, and prior studies examining online volunteer posting data have also been limited by a lack of data on actual volunteer commitments (Penner et al., 2005). Therefore, further research is needed that quantifies the amount of formal and informal volunteering in response to COVID-19. Fourth, given that individuals do not register to visit a post website, a single individual may have contributed to multiple views across posts.

## Conclusions

Large increases in COVID-19-related, remote, and food distribution roles demonstrate volunteers were responsive to the needs of the community. However, decreases in the demand for volunteers highlight decreased opportunities for individuals to volunteer, which may contribute to unintended negative impacts on individuals, and operational challenges faced by NPOs which may limit their ability to provide services to those in need. Our findings demonstrate that crises can have long-lasting impacts on the mix of volunteer positions and organizations’ recruitment and that while demand for crisis-related volunteers may remain high over longer periods of time, sustaining interest in volunteers may be challenging.

## Supplemental Material

sj-docx-1-nvs-10.1177_08997640231163782 – Supplemental material for COVID-19 Pandemic, Physical Distancing Policies, and the Non-Profit Sector Volunteer Force Click here for additional data file. Supplemental material, sj-docx-1-nvs-10.1177_08997640231163782 for COVID-19 Pandemic, Physical Distancing Policies, and the Non-Profit Sector Volunteer Force by Michael Lebenbaum, Claire de Oliveira, Joanne McKiernan, France Gagnon and Audrey Laporte in Nonprofit and Voluntary Sector Quarterly
