# Supplementary material for: COVID-19 Pandemic, Physical Distancing Policies, and the Non-Profit Sector Volunteer Force
Source: Nonprofit Volunt Sect Q. 2023 Apr 19;53(1):274–88. doi: 10.1177/08997640231163782 (PMC10116199; doi:10.1177/08997640231163782)
Supplement: sj-docx-1-nvs-10.1177_08997640231163782 – Supplemental material for COVID-19 Pandemic, Physical Distancing Policies, and the Non-Profit Sector Volunteer Force [file sj-docx-1-nvs-10.1177_08997640231163782.docx]

Supplementary Material

Online Appendix Figure 1

A) Weekly average of total number of postings by type of position^a,b^

B) Weekly average of total rate of posting views by type of position^a^

Legend: ^a^Each of the background grey shades refers to a different period of physical distancing with the order being baseline, 1^st^ lockdown, stage 2, stage 3 (least restrictive), modified stage 2, 2^nd^ lockdown; ^b^LTC = Long-term care; *One week had a rate of 149.2

Online Appendix Table 1. Dates, brief description and link to more information on each physical distancing period

| Period/Policy | Date Initiated | Brief description | Link |
| --- | --- | --- | --- |
| First COVID-19 Case in Toronto/Ontario | January 25, 2020 | NA | https://www.ctvnews.ca/canada/novel-coronavirus-in-canada-here-s-a-timeline-of-covid-19-cases-across-the-country-1.4829917 |
| Baseline | February 3, 2020 | First day of data collection,  No physical distancing restrictions | NA |
| Lockdown | March 11, 2020 | Gathering limit set to 5 people, closures of non-essential businesses, city services, etc. | https://globalnews.ca/news/6671913/closed-cancelled-toronto-coronavirus-covid-19/  https://globalnews.ca/news/6746181/ontario-ban-gatherings/ |
| Stage 2 | June 24, 2020 | Gathering limit set to 10 people, re-opening of non-essential businesses, recreational and cultural spaces | https://www.toronto.ca/news/city-of-toronto-enters-stage-2-of-the-provinces-reopening/ |
| Stage 3 | July 31, 2020 | Gathering limit set to 50 people indoors/100 people outdoors, lowest level of restrictions on businesses/city/cultural services | https://www.toronto.ca/news/city-of-toronto-now-in-stage-3-reopening/ |
| Modified stage 2 | October 10, 2020 | Gathering limit set to 10 people indoors/25 people outdoors, additional restrictions on businesses/city/cultural services | https://news.ontario.ca/en/release/58767/ontario-implementing-additional-public-health-measures-in-toronto-ottawa-and-peel-region |
| Lockdown | November 23, 2020 | Gathering limit set to 10 people outdoor, closures of non-essential businesses, restrictions on city/cultural services | https://www.toronto.ca/news/city-of-toronto-supports-provincial-lockdown-measures-to-reduce-covid-19-transmission/ |

Online Appendix Table 2. Original and revised (after combinations) posting categories

| Original Posting Category | Revised Posting Category |
| --- | --- |
| 1. COVID-19 Response | 1. COVID-19 Response |
| 2. Accessibility and Disabilities | 2. Accessibility, Advocacy, Policy & Settlement |
| 3. Advocacy, Policy, and Research | 2. Accessibility, Advocacy, Policy & Settlement |
| 4. Settlement and Newcomers | 2. Accessibility, Advocacy, Policy & Settlement |
| 5. Administration and Customer Service | 3. Administration and Customer Service |
| 6. Arts and Culture | 4. Arts, Culture, Sports & Recreation |
| 7. Sports and Recreation | 4. Arts, Culture, Sports & Recreation |
| 8. Boards, Committees, and Leadership | 5. Boards, Committees, and Leadership |
| 9. Children and Youth | 6. Children and Youth, Education and Literacy |
| 10. Education and Literacy | 6.Children and Youth, Education and Literacy |
| 11. Environment and Animals | 7. Environment and Animals |
| 12. Event Assistance and Planning | 8. Event Assistance and Planning |
| 13. Marketing, Communications, and Fundraising | 9. Marketing, Communications, and Fundraising |
| 14. Driving and Transportation | 10. Poverty Reduction, Social Services, Food Distribution and Driving |
| 15. Social Services and Poverty Reduction | 10. Poverty Reduction, Social Services, Food Distribution and Driving |
| 16. Food Preparation and Kitchen Support | 10. Poverty Reduction, Social Services, Food Distribution and Driving |
| 17. Health and Mental Health | 11. Seniors and Health |
| 18. Seniors and Long-Term Care | 11. Seniors and Health |
| 19. Suitable for Youth (14-17) | 12. Suitable for special populations |
| 20. Suitable for Families and Children | 12. Suitable for special populations |
| 21. Suitable for People with Mobility Devices | 12. Suitable for special populations |
| 22. Suitable for Groups | 12. Suitable for special populations |
| 23. Suitable for Court Ordered Volunteers | 12. Suitable for special populations |
| 24. Suitable for People Learning English | 12. Suitable for special populations |
| 25. IT Support | 13. Technical |
| 26. Trades and Maintenance | 13. Technical |

Online Appendix Table 3. Keywords used to search post titles to find specific types of volunteering

| Variable | Keywords |
| --- | --- |
| Event Assistance | Event; OR  Primary category = “Event Assistance and Planning” |
| Food | Meals on Wheels, Food Bank, Breakfast, Lunch, Dinner, Brunch, Meal, Kitchen, Cafe, Food, Chef, Cook, Feed, Dining, Coffee, Bread; OR  Primary category: “Food preparation and Kitchen Support” |
| Non-Food Driver-Mover | Driver, Delivery, Mover, Move, Transportation; OR  Primary category = “Driving and Transportation AND Not Food |
| Social or Mental Health | Caller, Calling, Chatter, Hotline, Drop-in, Greeter, Letter Writer, Telephone, Mental Health, Counselling, Distress, Counsellor, Counsel, Tele-Therapy, Support Line, Therapist |
| Support with Errands | Shopping, Shopper, Errand, Child Care, Grocery |
| Technology | IT, Tech/Technology/Technical Support, Web Developer; OR  Primary category = “IT Support” |
| Hospital/Long-term care | Hospital or Long-term care  Organization listed:   - https://www.toronto.ca/311/knowledgebase/kb/docs/articles/private/hospitals-toronto-and-gta-and-long-term-care-rehabilitation-facilities.html - https://www.torontocentralhealthline.ca/listservices.aspx?id=10665 |

Oline Appendix Table 4. Segmented regression analysis of the effect of physical distancing policies on the mean rate of daily views^a,b^

|  | Poisson regression | | Random Effects Poisson Regression | |
| --- | --- | --- | --- | --- |
|  | IRR^c^ | P-value | IRR^c^ | P-value |
| Changes in level (i.e., immediate change) |  |  |  |  |
| 1^st^ Lockdown (March 11-June 23) | 0.52 | <0.001 | 0.51 | <0.001 |
| Stage 2 (June 24-July 30) | 0.98 | 0.715 | 1.04 | 0.458 |
| Stage 3 (July 31-October 9) | 1.02 | 0.707 | 0.95 | 0.469 |
| Modified Stage 2 (October 10-November 22) | 0.94 | 0.242 | 0.97 | 0.649 |
| 2^nd^ Lockdown (November 23-January 4) | 1.02 | 0.625 | 0.98 | 0.681 |
| Changes in slope (i.e., gradual change) |  |  |  |  |
| Weeks since February 3^d^ | 1.00 | 0.009 | 0.99 | <0.001 |
| Weeks since start of 1^st^ Lockdown (March 11)^d^ | 1.14 | <0.001 | 1.10 | <0.001 |
| Weeks since start of Stage 2 (June 24)^d^ | 0.82 | <0.001 | 0.85 | <0.001 |
| Weeks since start of Stage 3 (July 31)^d^ | 1.02 | 0.409 | 1.07 | 0.045 |
| Weeks since start of Modified Stage 2 (October 10)^d^ | 0.89 | <0.001 | 0.93 | 0.010 |
| Weeks since start of 2^nd^ Lockdown (November 23)^d^ | 1.02 | 0.311 | 0.95 | 0.123 |

Legend: ^a^All models are run with robust standard errors; ^b^Model also controlled for days of the week, unemployment rate and weekly COVID-19 cases; ^c^IRR = Incidence Rate Ratio ^d^All time variables are modeled continuously as weeks since start of the period (and coded 0 prior to that day).
